# The GLP-1 Receptor Agonist Exenatide Ameliorates Bone Composition and Tissue Material Properties in High Fat Fed Diabetic Mice

**DOI:** 10.3389/fendo.2019.00051

**Published:** 2019-02-12

**Authors:** Sity Aishah Mansur, Aleksandra Mieczkowska, Peter R. Flatt, Daniel Chappard, Nigel Irwin, Guillaume Mabilleau

**Affiliations:** ^1^School of Biomedical Sciences, University of Ulster, Coleraine, United Kingdom; ^2^Groupe études remodelage osseux et biomatériaux, GEROM, SFR 42-08, Université d'Angers, Institut de Biologie en Santé, CHU d'Angers, Angers, France; ^3^Service commun d'imageries et d'analyses microscopiques, SCIAM, SFR 42-08, Université d'Angers, Institut de Biologie en Santé, CHU d'Angers, Angers, France; ^4^Bone Pathology Unit, Angers University Hospital, Angers, France

**Keywords:** exenatide, bone fragility, bone composition, type 2 diabetes, glucagon-like peptide-1

## Abstract

Type 2 diabetes mellitus (T2DM) has recently been recognized as a significant risk factor for bone fragility. Careful investigations of bone mechanical properties in human studies suggested possible alterations of bone composition, although this axis has poorly been investigated. The main aim of this study was to evaluate the impact of high fat diet-induced diabetes and therapy using the clinically approved GLP-1 receptor agonist, exenatide, on tissue bone mechanical properties and compositional parameters. Male mice had free access to high fat diet for 16 weeks to induce diabetes prior to commencement of the study. Exenatide was administered twice daily by i.p. injection at a dose of 25 nmol/kg for 52 days. Normal and high fat diet fed (HFD) mice injected with saline were used as controls. Bone mechanical properties was assessed at the organ level by 3-point bending and at the tissue level by nanoindentation. Bone microarchitecture was investigated by microcomputed tomography and bone composition was evaluated by Fourier transform infrared imaging. HFD mice exhibited profound alterations of bone mechanical properties at both the organ and tissue level. Collagen maturity as well as trabecular and cortical bone microarchitectures were abnormal. Administration of exenatide, led to clear ameliorations in bone mechanical properties at the organ and tissue levels by modifications of both cortical microarchitecture and bone compositional parameters (collagen maturity, mineral crystallinity, carbonate/phosphate ratio, acid phosphate content). These results bring new light on the mode of action of exenatide in bone physiology and demonstrate the value of GLP-1 mimetics in the treatment of fragility fractures in diabetes.

## Introduction

Diabetes mellitus is now considered an independent risk factor for bone fragility fractures (1, 2). Patients with type 2 diabetes mellitus (T2DM) present with normal or slightly elevated values for bone mineral density, even when normalized for body mass index (3, 4). Paradoxically, T2DM is associated with an augmentation in the frequency of fragility fractures (5–7). The possible explanation for this elevated fracture occurrence may reside in T2DM-related complications (reduced muscle quality, retinopathy, nephropathy, poor balance and increased falls), abnormal glycemic control and anti-diabetic medications that may compromise bone quality (8), i.e., an ensemble of structural and compositional properties that are very important for bone mechanical properties (9). Indeed, an elegant study from Farr and colleagues using the OsteoProbe® microindentation device reported lower bone material mechanical properties index in post-menopausal T2DM patients as compared with controls (10). Taken together, these observations suggest alterations of bone in T2DM at the compositional levels. Compositional parameters, regrouping specific properties of the bone mineral and collagen, have already been correlated with mechanical strength and fracture risk (11) in osteoporosis but have scarcely been investigated in T2DM and even less so in response to anti-diabetic medications.

Glucagon-like peptide 1 (GLP-1) is a hormone produced by enteroendocrine L-cells, located primarily in the ileum and colon (12). GLP-1 is an incretin hormone that potentiates glucose-mediated insulin secretion by pancreatic beta cells. Previous studies have suggested that patients with T2DM have an attenuated response to endogenous GLP-1 and that infusion of exogenous synthetic GLP-1 could be considered a treatment option (13). As such, several GLP-1 mimetics have been developed and approved for the treatment of T2DM and randomized clinical trials demonstrated the superiority of GLP-1 receptor agonists to other antidiabetic drugs in reducing HbA1c and blood pressure, and initiating weight loss without hypoglycemia risk (14). Despite a relative high cost, GLP-1 receptor agonists are increasingly been prescribed to T2DM patients. Among all GLP-1 receptor agonists, exenatide was the first molecule approved for the treatment of T2DM in 2005. Exenatide is a synthetic derivative of exendin-4, a naturally occurring peptide found in salivary secretion of the Gila monster lizard, with a 53% amino acid sequence overlap with human GLP-1 (15). Exenatide has a longer duration of action and similar receptor binding and cAMP release properties than endogenous GLP-1 (16).

Meta-analysis of randomized clinical trials or observational studies, although supporting a beneficial role of GLP-1 receptor agonists in reducing HbA1c levels, reported divergent conclusions on the beneficial actions of GLP-1 analogs in reducing fracture risk (17–20). However, these studies presented several limitations that may hamper skeletal interpretation. In rodent models, the use of GLP-1 receptor agonists augmented bone mass and ameliorated bone structural properties in ovariectomy-induced bone loss (21–24) or genetically inherited T2DM models (25–27). However, despite the fact that GLP-1 receptor deficient animals present with alterations of bone composition (28), little is known about the action of GLP-1 receptor agonists on bone compositional parameters in T2DM, although changes in collagen and mineral properties have been suspected to play a role in the etiology of fragility fractures in T2DM.

Therefore, the aims of the present study were (i) to evaluate whether the high-fat diet (HFD) diabetic mouse model presents with alterations of bone composition and (ii) to ascertain whether the use of a GLP-1 receptor agonist, exenatide, improves skeletal health in this model.

## Materials and Methods

### Reagents

Exenatide (Ex) was purchased from GL Biochem Ltd. (Shangai, China). Purity and molecular mass were verified by high performance liquid chromatography and matrix-assisted laser desorption/ionization-time of flight mass spectrometry. All other chemicals were obtained from Sigma-Aldrich (Lyon, France) unless otherwise stated.

### Animals

**Figure 1:**
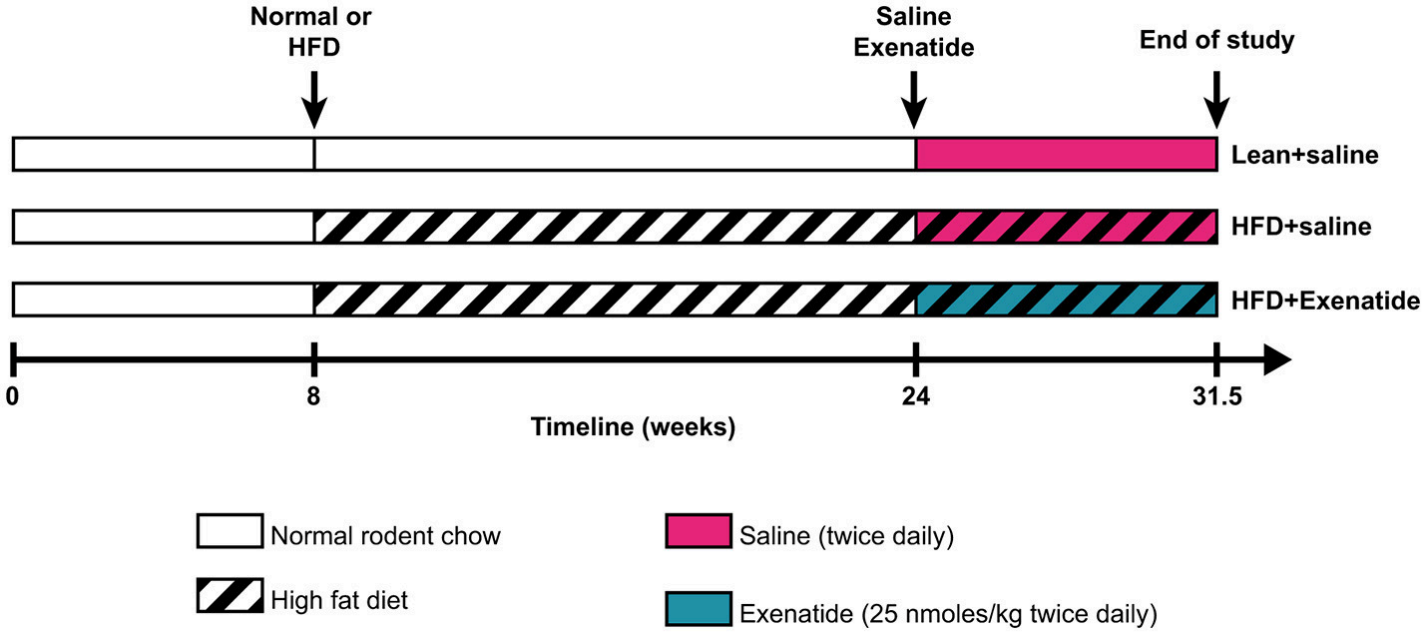
Schematic representation of the experimental design used in this study.

Male NIH Swiss mice (Hsd:NIHS) were obtained from Envigo Ltd (Blackthorn, UK) at 8 weeks of age. Animals were individually housed in an air-conditioned room at 22 ± 2°C with a 12-h light/dark cycle, and were provided with tap water and high fat diet (45% fat, 20% protein, 35% carbohydrate for a total of 26.2 kJ/g; Special Diet Service, Essex, UK) *ad libitum*. In an unpublished pilot study, we observed that after 112 days on high fat diet, mice exhibited significantly increased body weight and hyperglycaemia (fasting glucose level >14 mmol/l) as well as a 40% reduction in trabecular bone mass and a 27% reduction in cortical thickness at the femur. High fat diet diabetic (HFD) mice were then divided into two matched groups (*n* = 8) that received twice daily saline (0.9% NaCl – HFD + saline) or exenatide (25 nmol/kg bw – HFD + Ex) intraperitoneally for 52 days. This protocol was chosen based on positive metabolic and bone-associated effects observed using related peptide-based drugs with similar dosing regimens and animal models (29, 30). Normal male mice (*n* = 8), fed a standard rodent diet (10% fat, 30% protein and 60% carbohydrate for a total of 13 kJ/g; Trouw Nutrition, Northwich, UK) and injected twice daily with saline were used as controls (Lean + saline). Figure 1 represents a schematic of the experimental design. All procedures were conducted according to UK Home Office Regulations (UK Animals Scientific Procedures Act 1986) and approved by the Ulster University Institutional animal care committee. At the end of the study, body weight, average daily food intake, non-fasting plasma glucose and insulin levels, glucose tolerance test and insulin sensitivity were assessed as reported previously (31). Bone mineral density (BMD, g/cm^2^) and total body fat (g) were measured with a Lunar PIXImus scanner (Inside Outside Sales, Wisconsin, U.S.A.) as described previously (30). At necropsy, femurs and tibias were cleaned of soft tissues and stored in 70% ethanol until used, as described below.

### MicroCT

MicroCT analyses were performed with a Skyscan 1172 microtomograph (Bruker MicroCT, Kontich, Belgium) operated at 70 kV, 100 μA, 340-ms integration time. The isotropic pixel size was fixed at 4 μm, the rotation step at 0.25° and exposure was done with a 0.5-mm aluminum filter. To calibrate for bone mineral density, hydroxyapatite (HA) rods (2-mm diameter, at hydroxyapatite densities of 0.25 and 0.75 gHA/cm^3^) and volumes of saline and air were also scanned with the same parameters. Each 3D reconstruction image dataset was binarized using global thresholding. Trabecular volume of interest (VOI) was located at the proximal tibia, 0.5 mm below the growth plate and extended 2 mm down. Using the hydroxyapatite calibration scans, the grayscale dataset was transformed into Hounsfield units (HU) by assigning an HU value of 0 to the average grayscale index of saline, and an HU value of −1,000 to black pixels (air). Next, average HU values for the two mineralized phantoms (0.25 and 0.75 gHA/cm^3^) were used to create a linear relationship between HU and mineral density.The threshold value to discriminate mineralized tissue from bone marrow was set at 0.4 g/cm^3^ and resulting binarized images were compared visually with original images. All microCT parameters were determined according to guidelines and nomenclature proposed by the American Society for Bone and Mineral Research (32).

### Bone Histomorphometry

After microCT, tibias were embedded, undecalcified in methylmethacrylate at 4°C to preserve enzyme activities. Sagittal sections (7-μm thickness) were performed on a heavy duty microtome equipped with a 50° tungsten carbide knife. Four non-serial sections were stained with toluidine blue for the measurement of bone marrow adiposity and the number of osteoblasts. Four additional sections were stained for the osteoclastic tartrate resistant acid phosphatase (TRAcP). Four sections were stained with Goldner Trichrome. Regions of interest were located 0.5 mm below the growth plate and extended 3 mm down. Standard bone histomorphometrical nomenclatures, symbol and units were used as described in the report of the American Society for Bone and Mineral Research (33).

### Bone Mechanical Properties Assessment

Three-point bending experiments were performed on femurs after rehydrating bones at 4°C for 24 h. This test investigates the overall resistance of the whole bone that depends from both the structural and the compositional properties and represents the strength at the organ level. Measurements were done with an Instron 5942 (Instron, Elancourt, France) as reported previously (34). Briefly, the span length was fixed at 10 mm and femurs were positioned horizontally with the anterior face downward. The press head was applied vertically to the midshaft with a loading speed of 2 mm/min until failure. The load-displacement curve was acquired with the Bluehill 3 software (Instron). Ultimate load, ultimate displacement, stiffness and total absorbed energy were computerized (35). After three-point bending experiments, distal portions of femur were embedded undecalcified in polymethylmethacrylate at 4°C and cross-sections were made at the midshaft using a diamond saw (Accutom, Struers, Champigny sur Marne, France). Cross-sections were polished to a 1-μm finish with diamond particles (Struers, France) and subjected to rehydration in saline 24 h prior to nanoindentation testing. Nanoindentation testing is independent of the overall bone microstructure and hence represents the strength of the bone matrix at the tissue level. Twelve indentations were randomly made in cortical bone with a NHT-TTX system (Anton Paar, Les Ulis, France) at distance from osteocyte lacuna and microcracks. Loading/unloading was performed at a rate of 40 mN/min up to a depth of 900 nm. At maximum load, a holding period of 15 s was applied to avoid creeping of the bone material. The following material properties at the tissue-level, maximum load (Force max), indentation modulus (E_IT_), hardness (H_IT_) and dissipated energy (W_plast_), were determined according to Oliver and Pharr (36).

After nanoindentation, cross-sections were stained with toluidine blue and cortical thickness and cross-sectional moment of inertia alongside the antero-posterior axis were computerized.

### Fourier-Transform Infrared Imaging (FTIRI)

Cross-sectional sections (4 μm) of the midshaft femur were sandwiched between barium fluoride optical windows. Cross-sections were divided into four quadrants (anterior, posterior, lateral and medial) and all subsequent infrared acquisitions and analyses have been performed at the posterior quadrant. FTIRI was performed with a vertex 70 spectrometer (Bruker, Ettlingen, Germany) interfaced with a Hyperion 3,000 microscope and a focal plane array detector (64 × 64 pixels) covering a field of view of 180 × 180 μm. Nine field-of-view were stitched together to allow sufficient bone to be analyzed. Sections were scanned with a spectral resolution of 8 cm^−1^ (spectral region 900–2,000 cm^−1^). Each spectrum was corrected for Mie scattering with the RMieS-EMSC_v5 algorithm (kind gift of Prof Peter Gardner, University of Manchester, UK) prior to be subjected to pMMA substraction. Evaluation of spectral images was done with a lab-made routine script in Matlab R2016b (The Mathworks, Natick, MA) as previously described (37). FTIR bone parameters (38) calculated were: (1) mineral/matrix ratio (area of v1, v3 phosphate/area amide1); (2) acid phosphate content (intensity ratio 1,127cm^−1^/1,096 cm^−1^) (39); (3) mineral crystallinity (intensity ratio 1,030 cm^−1^/1,020 cm^−1^), reflecting crystal size and perfection; and (4) collagen maturity (intensity ratio 1,660 cm^−1^/1,690 cm^−1^). The carbonate/phosphate ratio (intensity v3 carbonate located at ~1,415 cm^−1^/1,030 cm^−1^) was computed after substracting the organic matrix spectrum (40). For each of the compositional parameters, the mean and full width at half maximum of the pixel distribution (excluding the zero background values) were computed and represented as mean and heterogeneity.

### Statistical Analysis

Mechanical, structural, histomorphometrical and compositional data were analyzed using Prism 6.0 (GraphPad Software Inc, La Jolla, CA). Due to the adaptive nature of bone and an observed higher body mass in HFD + saline animals, *in vivo* data were adjusted for body-size (body mass × femur length) using a linear regression method as reported in detail elsewhere (41). An analysis of variance (ANOVA) was employed to test for significance in any of the studied parameters. The normality in data distribution was tested with the Brown-Forsythe test. The association between mechanical properties, plasma glucose level, cortical bone microarchitecture and bone compositional parameters were investigated by stepwise multiple regression analysis with a probability estimate of 0.15 and a tolerance of 0.1 using Systat 13 (Systat Software Inc., San Jose, CA). Multiple linear regression models were constructed, making the dependent variable a function of non-fasting plasma glucose levels, collagen maturity, phosphate/amide ratio, mineral crystallinity, carbonate/phosphate ratio and acid phosphate content. Differences at p equal or less than 0.05 were considered significant.

## Results

### HFD Animals Exhibit a Higher Body Mass but Lower Bone Mineral Densities

As presented in Table 1, body and fat masses, non-fasting glucose and glucose tolerance levels were significantly increased in the HFD + saline group as compared with lean + saline animals. Administration of exenatide in HFD mice had no effect on food intake as well as body and fat masses but significantly reduced non-fasting glucose levels by 59% (*p* < 0.001) and augmented non-fasting insulin levels by 84% (*p* = 0.003). In addition, exenatide significantly improved glucose tolerance (*p* = 0.05) and peripheral insulin sensitivity (*p* = 0.05) when compared to saline treated HFD mice (Table 1). Bone mineral density assessed either for the whole body or at lumbar or femur sites revealed significant lower values in HFD + saline mice as compared with lean + saline animals (Table 1). On the other hand, administration of exenatide in HFD mice did not ameliorate bone mineral density at any site.

**Table 1:** Effects of HFD-induced diabetes and treatment with exenatide on metabolic and compositional parameters.

	**Lean + saline**	**HFD + saline**	**HFD + Ex**
Daily food intake (g)	4.2 ±0.3	3.9 ±0.3	3.6 ±0.2
Body mass (g)	43 ±2^***^	58 ±3	54 ±1^$$^
Fat mass (%)	26 ±2^*^	34 ±2	34 ±1^$^
Non-fasting glucose (mM)	4.9 ±0.2^***^	22.5 ±2.7	9.3 ±2.2^***^
Non-fasting insulin (mM)	1.8 ±0.3	2.5 ±0.4	4.6 ±0.5^**^^,^ ^$$$^
Glucose tolerance (AUC_0−60min_) (mM.min)	719 ±73^**^	1,043 ±102	810 ±25^*^
Insulin sensitivity (AAC_0−60min_) (%change plasma glucose. min)	2,192 ±130^***^	1,166 ±127	1,829 ±155^*^
Whole body bone mineral density (mg/cm^2^)	72 ±1^***^	53 ±1	59 ±1^$^
Lumbar bone mineral density (mg/cm^2^)	72 ±3^***^	54 ±2	60 ±2^*^
Femur bone mineral density (mg/cm^2^)	115 ±3^**^	91 ±3	102 ±4^*^

*For glucose tolerance tests, blood glucose concentrations were measured before and 15, 30, and 60 min after i.p injection of glucose (18 mmol/kg), with 0–60 min AUC values noted in Table. For peripheral insulin sensitivity, blood glucose concentrations were measured before and 15 and 60 min after i.p injection of insulin (10 U/kg), with 0–60 min AAC values noted in Table. Glucose tolerance tests were conducted in mice fasted for 18 h whereas insulin sensitivity tests were conducted in non-fasted mice*.

* *p ≤ 0.05*,

** *p < 0.01*,

*** *p < 0.001 vs. HFD + saline*.

$ *p ≤ 0.05*,

$$ *p < 0.01*,

$$$ *p < 0.001 vs. Lean + saline. Values are means ± SEM for 8 mice*.

### Administration of Exenatide Ameliorated Bone Mechanical Properties at the Organ Level

Next, we thought it prudent to ascertain biomechanical resistance of bone tissue at both the organ and tissue levels. As represented in Table 2, HFD + saline animals presented with a compromised bone mechanical properties at the organ level as suggested by significant lower values for all mechanical parameters investigated. Administration of exenatide ameliorated bone mechanical properties at the organ level in HFD mice as revealed by significant augmentations in all mechanical parameters.

**Table 2:** Effects of HFD-induced diabetes and treatment with exenatide on bone mechanical response at the organ and tissue levels.

	**Lean + saline**	**HFD + saline**	**HFD + Ex**
**ORGAN LEVEL**			
Ultimate load (N)	34.7 ±1.5^***^	25.2 ±1.3	30.8 ±1.3^**^
Yield load (N)	26.1 ±1.4^***^	18.7 ±1.0	22.8 ±1.2^*^
Ultimate displacement (mm)	0.48 ±0.04^***^	0.24 ±0.01	0.33 ±0.02^*^^,^ ^$$$^
Post-yield displacement (mm)	0.30 ±0.04^***^	0.10 ±0.01	0.18 ±0.02^*^^,^ ^$$^
Stiffness (N/mm)	177 ±10^***^	121 ±7	152 ±8^*^^,^ ^$^
Work-to-failure (N.mm)	9.5 ±1.0^***^	4.2 ±0.5	7.0 ±0.5^**^^,^ ^$^
**TISSUE LEVEL (3-POINT BENDING)**			
Ultimate stress (MPa)	237 ±12^***^	164 ±10	189 ±8^$$^
Yield stress (MPa)	177 ±9^***^	122 ±8	139 ±8^$^
Ultimate strain	0.050 ±0.004^***^	0.025 ±0.002	0.035 ±0.002^*^^,^ ^$$^
Post-yield strain	0.031 ±0.004^***^	0.011 ±0.001	0.020 ±0.002^*^^,^ ^$^
Elastic modulus (GPa)	11.8 ±0.8^***^	7.6 ±0.5	8.8 ±0.5^$$^
**TISSUE LEVEL (NANOINDENTATION)**			
Maximum force (mN)	17.7 ±0.6^***^	11.4 ±0.8	12.8 ±0.6^$$$^
Indentation modulus (GPa)	18.9 ±0.4^***^	12.9 ±0.5	13.8 ±0.5^$$$^
Indentation hardness (MPa)	980 ±22^***^	605 ±24	759 ±17^***^^,^ ^$$$^
Work of indentation (pJ)	4,285 ±157^***^	2,837 ±141	3,000 ±138^$$$^

* *p < 0.05*,

** *p < 0.01*,

*** *p < 0.001 vs. HFD + saline*.

$ *p < 0.05*,

$$ *p < 0.01*,

$$$ *p < 0.001 vs. Lean + saline. Values are means ± SEM for 8 mice*.

### Administration of Exenatide Improved Also Mechanical Properties at the Tissue Level

Bone mechanical properties have been investigated at the tissue level by two methods. The first one used equations from engineering beam theory has a first estimate (Table 2). Ultimate stress, yield stress, ultimate strain, post-yield strain and elastic modulus were all reduced in HFD + saline animals as compared with lean + saline mice. Administration of exenatide, only improved significantly ultimate strain and post-yield strain. Bone mechanical properties have also been investigated by nanoindentation in femur cortical bone. As compared with lean + saline, maximum force, indentation modulus, indentation hardness and work of indentation were significantly reduced in HFD + saline animals. Administration of exenatide in these animals, only ameliorated the hardness of the bone matrix by 25% (*p* < 0.001).

### Administration of Exenatide Ameliorated the Compromised Trabecular Bone Microarchitecture Observed in HFD Mice

**Figure 2:**
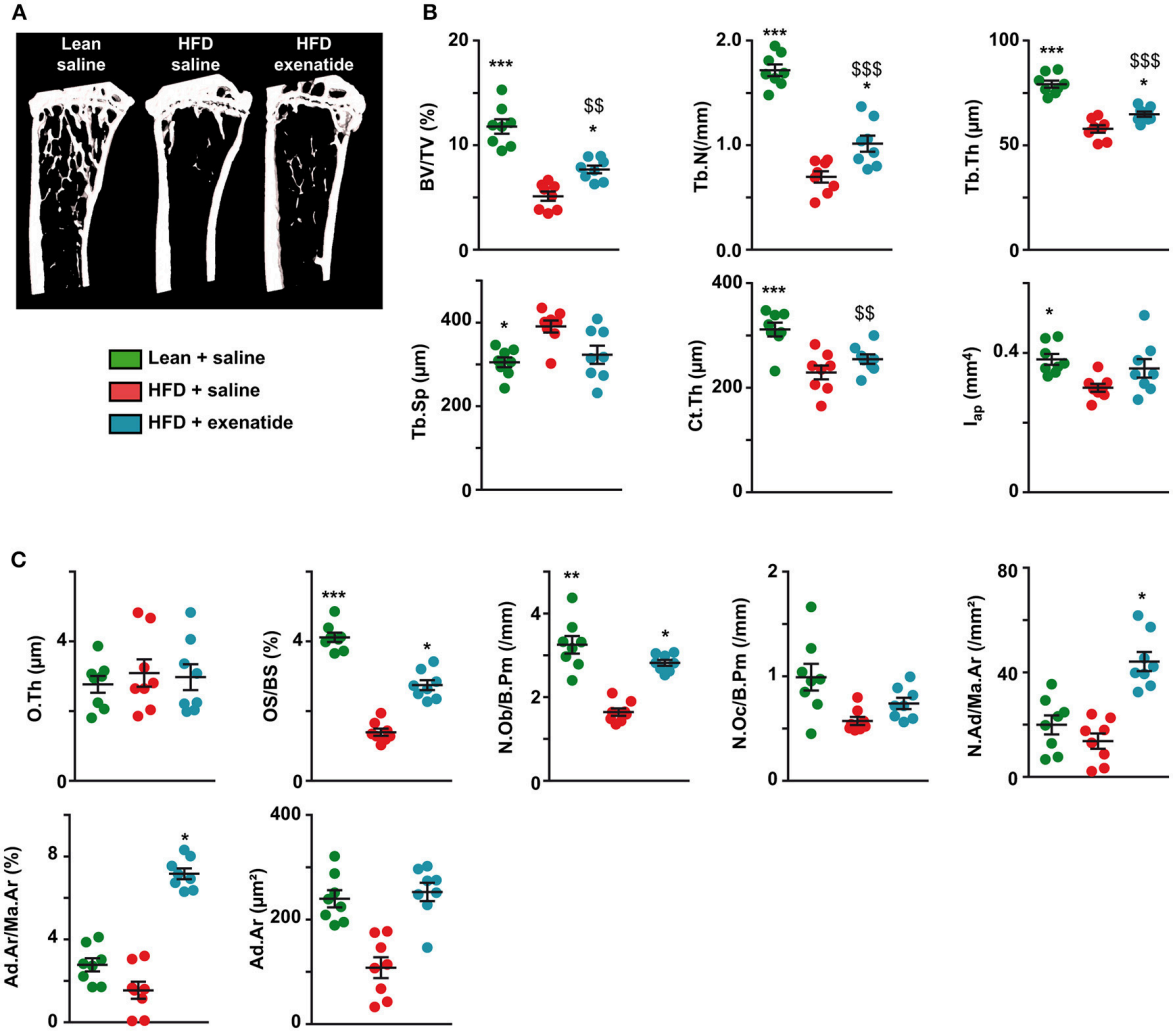
Effects of HFD-induced diabetes and treatment with exenatide on trabecular and cortical bone microarchitectures. **(A)** Three-dimensional models of tibias were reconstructed and highlight differences in trabecular bone network. **(B)** Trabecular microarchitecture parameters determined at the proximal tibia and cortical microarchitecture parameters measured at the midshaft femur. BV/TV: trabecular bone volume, Tb.N: trabecular number, Tb.Th: trabecular thickness, Tb.Sp: trabecular spacing, Ct.Th: cortical thickness and Iap: cross-sectional moment of inertia about the anteroposterior axis. ^*^p < 0.05, ^**^p < 0.01, ^***^p < 0.001 vs. HFD + saline. ^$^*p* < 0.05, ^$$^*p* < 0.01, ^$$$^*p* < 0.001 vs. Lean + saline. Values are means ± SEM for 8 mice. **(C)**, Histomorphometrical parameters measured at the proximal tibia. O.Th, osteoid thickness; OS/BS, osteoid surface; N.Ob/B.Pm, number of osteoblasts; N.Oc/B.Pm, number of osteoclasts; N.Ad/Ma.Ar, adipocyte number; Ad.Ar/Ma.Ar, bone marrow adiposity volume; Ad.Ar, adipocyte area. ^*^p < 0.05, ^**^p < 0.01, ^***^p < 0.001 vs. HFD + saline. ^$$^*p* < 0.01, ^$$$^*p* < 0.001 vs. Lean + saline.

As bone mechanical properties at the organ level depends on bone structural and bone compositional properties, we next investigated trabecular and cortical bone microarchitectures. Figure 2A represents 3D models of coronal sections of tibias. HFD + saline animals seems to exhibit lower trabecular bone in the proximal tibia metaphysis, while administration of exenatide increases this parameter. Indeed, careful investigation of trabecular microarchitecture (Figure 2B) highlighted that, as compared with the lean + saline group, HFD + saline animals presented with lower trabecular bone volume. Alterations of the trabecular network were also evident and represented by reductions in trabecular number and trabecular thickness, and augmentation in trabecular spacing. Administration of exenatide significantly augmented trabecular bone volume and improved the connectivity of the trabecular network by significantly increasing both trabecular number and thickness. Although a tendency to lower values for Tb.Sp were observed in HFD + Ex mice, this parameter did not reach statistical significance (*p* = 0.084).

Furthermore, cortical bone microarchitecture was also modified in HFD + saline group compared with lean + saline animals and represented by lower values for cortical thickness and cross-sectional moment of inertia about the anteroposterior axis. Administration of exenatide in HFD animals did not result in significant improvements in any of these cortical parameters.

At the matrix and cellular levels, osteoid thickness was not different between the three groups of animals suggesting that initiation of bone mineralization was not altered. Osteoid surfaces were significantly lowered in HFD + saline mice as compared with lean + saline controls. Administration of exenatide significantly augmented osteoid surfaces suggesting new bone formation. This is even corroborated by the number of osteoblasts that was reduced in HFD + saline animals and augmented after administration of exenatide. No modifications in the number of osteoclasts was observed between the three groups of animals. However, bone marrow adiposity parameters were not significantly different between lean + saline and HFD + saline animals (Figure 2C). On the other hand, the administration of exenatide in HFD animals led to significant augmentations of adiposity volume and number of adipocytes in the bone marrow. The number of osteoclasts was not significantly different between the three groups of animals. On the other hand, the number of osteoblasts was significantly reduced in HFD + saline animals (−50%, *p* = 0.003) as compared with lean animal. The administration of exenatide led to a significant augmentation in this parameter.

### Administration of Exenatide Modified Bone Compositional Parameters in HFD Mice

**Figure 3:**
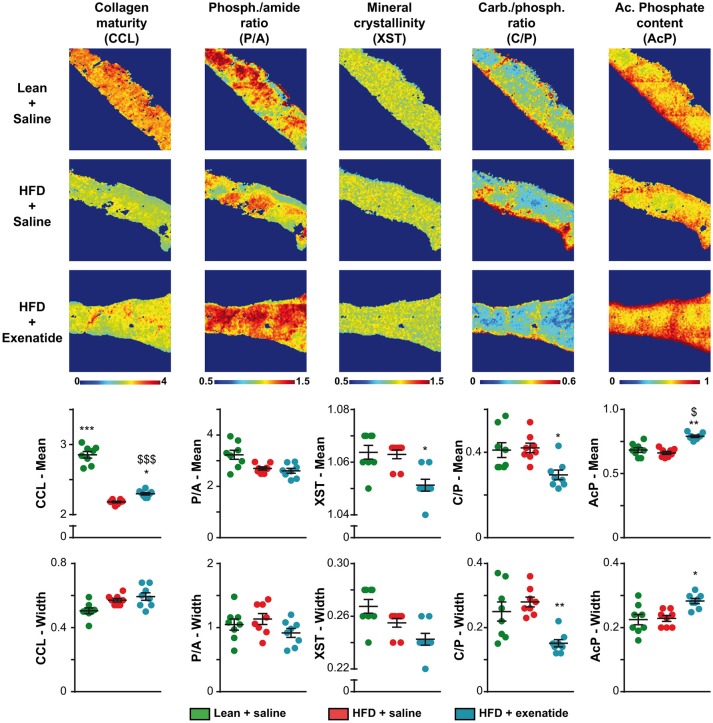
Effects of HFD-induced diabetes and treatment with exenatide on bone compositional parameters. Bone composition parameters were assessed by Fourier transform infrared imaging (FTIRI) at the posterior quadrant of the femur. Some FTIRI images, representing the overall stitching of nine consecutive field-of-views, are presented. The pseudo-color represents the degree of each parameter overall the full width of the cortical bone. The mean and width at half maximum, representing the mean and heterogeneity, respectively, of each parameter was also investigated and represented below FTIRI images. Values are means ± SEM for 8 mice. ^*^p < 0.05, ^**^p < 0.01, ^***^p < 0.001 vs. HFD + saline. ^$^*p* < 0.05, ^$$$^*p* < 0.001 vs. Lean + saline.

Next, as bone composition has been suspected to be altered in T2DM, we investigated compositional parameters over the full cortical width by infrared imaging. Figure 3 represents infrared images over the cortical width of the lean + saline, HFD + saline, and HFD + Ex groups. Some differences in the pixel intensity distribution in the bone matrix were obvious especially for collagen maturity, carbonate/phosphate ratio and acid phosphate content. Indeed, as compared with lean + saline animals, HFD + saline mice exhibited a significant 23% reduction (*p* < 0.001) in the mean collagen maturity whilst heterogeneity was not significantly different. None of the other compositional parameters, investigating mostly the mineral composition and crystallinity, were different between the two groups of animals. Administration of exenatide to HFD mice led to a significant augmentation in the mean collagen maturity. Significant lower values for the mean crystallinity as well as mean and heterogeneity of carbonate/phosphate ratio were also observed with exenatide treatment. In contrast, the mean and heterogeneity of acid phosphate content presented with significant higher mean values.

### Contribution of Changes in Plasma Glucose Levels, Microarchitectural and Compositional Parameters to Mechanical Property of Cortical Bone

In order to decipher the possible association between mechanical outcome and plasma glucose levels, microarchitectural and compositional parameters, we constructed several multiple regression models summarized in Table 3. Ultimate load was inversely associated with plasma glucose when saline-treated animals only were considered. However, in HFD animals, ultimate load depended greatly of carbonate/phosphate ratio and weakly to plasma glucose. Ultimate displacement was only associated with collagen maturity in saline-treated animals. In HFD animals, this mechanical parameter was greatly associated with mineral crystallinity, mildly with acid phosphate content and collagen maturity, and weakly with plasma glucose. In saline-treated animals, stiffness was associated only with cortical thickness. In HFD animals, stiffness was associated with acid phosphate content and carbonate/phosphate ratio. However, it is worth noting that although these two regression models were statistically significant, the adjusted *R*^2^ was relatively poor (0.336–0.481). In saline-treated and HFD-treated animals, post-yield displacement was linked to collagen maturity. Work to fracture was only associated with collagen maturity in saline-treated animals with a relatively good adjusted *R*^2^ of 0.761. In HFD animals, work-to-fracture was associated strongly with mineral crystallinity, mildly with acid phosphate content and collagen maturity and weakly with plasma glucose.

**Table 3:** Multiple regression analyses of the relations between mechanical properties, plasma glucose level, cortical bone microarchitecture, and bone compositional parameters.

**Dependent variable**	**Groups**	**Model adjusted *R*^2^**	**Model *p*-value**	**Parameter**	**β**	**Parameter *p*-value**
Ultimate load	Saline-treated	0.650	<0.001	Plasma glucose	−0.556	<0.001
	HFD	0.507	0.017	Plasma glucose	−0.697	0.007
				Carbonate/phosphate ratio	−27.737	0.039
Ultimate displ.	Saline-treated	0.903	<0.001	Collagen maturity	0.630	0.001
	HFD	0.824	0.002	Plasma glucose	−0.011	0.010
				Collagen maturity	0.726	0.013
				Mineral crystallinity	−6.430	0.019
				Acid phosphate content	0.855	0.012
Stiffness	Saline-treated	0.336	0.011	Ct.Th	0.411	0.011
	HFD	0.481	0.021	Acid phosphate content	368.2	0.009
				Carbonate/phosphate ratio	148.85	0.010
PYD	Saline-treated	0.611	0.002	Collagen maturity	0.212	0.002
	HFD	0.433	0.012	Collagen maturity	0.499	0.012
Work-to-fracture	Saline-treated	0.761	<0.001	Collagen maturity	9.073	<0.001
	HFD	0.729	0.008	Plasma glucose	−0.357	0.022
				Collagen maturity	22.5	0.033
				Mineral crystallinity	−166.2	0.035
				Acid phosphate content	21.403	0.048

## Discussion

Although patterns of T2DM differ by ethnic group and lifestyle, refined carbohydrates and saturated fat, which contribute to fat deposition in peripheral tissues and weight gain, are recognized important risk factors for development of T2DM. Indeed, overweight and obesity have been estimated to account for about 65–80% of new cases of T2DM (42). Patients with type 2 diabetes mellitus have an increased risk of bone fractures (1, 2). However, the exact underlying mechanism linking T2DM to higher fracture risk is poorly characterized. In the present study, we investigated changes in bone mechanical properties plus bone microarchitectural and compositional parameters in NIH swiss mice fed a high fat diet. In opposition to C57BL/6 mice, the NIH swiss model develop a frank fasting hyperglycemia (>14 mmol/L) over 112 days of high fat feeding and represents a validated model of obesity-induced T2DM (43). The potential effects of sustained treatment with exenatide, a GLP-1 receptor agonist drug already approved for the treatment of T2DM, in restoring bone mechanical properties was also investigated.

We found that HFD led to reduction of bone mineral density at all studied sites, and that this was associated with compromised bone mechanical properties not only at the organ but also at the tissue level, suggesting possible alterations of structural and compositional properties. Indeed, alterations of trabecular and cortical bone microarchitectures, bone cell count and compositional properties were undoubtedly evidenced. Administration of exenatide in this diabetic murine model led to significant improvement in all mechanical parameters at the organ level as well as amelioration in trabecular (BV/TV, Tb.N, Tb.Th) microarchitectures. Similar observations have been observed by others previously in several animal models of type 2 diabetes mellitus (25–27, 44). However, although a controversy exist on the presence of a functional GLP-1 receptor in bone cells, it is challenging to ascertain whether amelioration in trabecular microarchitecture, mechanical competency and changes in bone composition are due to direct effects on bone cells or indirect effects. Previously, in the db/db mouse model, we found that exenatide was capable of restoring impaired hindlimb blood flow that potentially could participate to better trabecular microarchitecture and bone formation (27).

Poor glycemic control is also an important factor suspected to act in the etiology of bone fracture in diabetes. Marked hyperglycemia was observed in the present study and administration of exenatide, as expected, reduced plasma glucose level. Multiple regression analyses revealed that although reduction in plasma glucose level participated in improvement in bone mechanical properties, its contribution was relatively modest and it did not appear as the main contributing factors. However, these results are to be weighted by the fact that reducing hyperglycemia might affect bone cell physiologies by reducing chronic inflammation and accumulation of advanced glycation end-products and hence might influence mineral deposition but also collagen cross-linking. As such, the contribution of lower plasma glucose levels to bone mechanical properties might have been higher than detected by multiple regression analyses.

The originality of our study resides in the investigation of compositional parameters as to the best of our knowledge; this has not yet been assessed previously and might provide an explanation for the decline in bone mechanical properties at the organ and tissue levels. The multiple regression analyses highlighted the important role of bone compositional parameters in bone mechanical properties as collagen maturity was a strong contributor to bone mechanical properties in saline treated animals (β between 0.212 and 9.73). In HFD animals, here again, collagen maturity, mineral crystallinity, carbonate/phosphate ratio and acid phosphate content were strong predictors of bone mechanical properties. Apart for bone stiffness, the high values of model adjusted *R*^2^ suggested a good agreement between experimental data and regression model. Furthermore, the energy required to fracture bone in three-point bending, and estimated as work-to fracture, was clearly dependent on compositional parameters rather than plasma glucose levels. Collagen maturity is a parameter reflecting the ratio of enzymatic mature (pyridinoline) over immature (dehydrodihydroxylysinonorleucine) collagen cross-links. However, collagen cross-linking is the result of enzymatic (lysine hydroxylase and lysine oxidase) and non-enzymatic, AGE driven, intermolecular bridging of collagen fibrils. The intermolecular cross-linking of bone collagen is a chemical feature that is intimately related to the way matrix collagen molecules are arranged in fibrils and provides fibrillary matrices with important mechanical properties such as tensile mechanical properties and viscoelasticity (45, 46). Non-enzymatic collagen cross-linking is represented by the accumulation of AGEs in the organic matrix and measurable by FTIRI (47). However, the required EDTA treatment led to very fragile bone sections and in the present study, more than 70% of our bone sections were not usable after EDTA treatment. This is unfortunate, as a reduction in AGE content could also have participated in ameliorating bone mechanical properties by directly affecting local mechanical response, but also by influencing the fate of bone cells.

Interestingly, exenatide administration resulted in reductions in mineral crystallinity, mean carbonate/phosphate ratio and its distribution heterogeneity and augmentation of acid phosphate content and heterogeneity. These mineral properties were unaffected by high fat diet and these observations suggest that the bone action of exenatide is beyond the simple reduction in plasma glucose levels and elevation in insulin level and sensitivity. However, it is worth noting that changes in mineral composition and crystallinity was not previously observed in the db/db mice model (27). Although in our study the administration of exenatide was for a longer period of time (~7.5 weeks vs. 4 weeks), this discrepancy might highlight differences in response to exenatide based on the etiology of type 2 diabetes (monogenic vs. lifestyle).

A limitation to this study is represented by the lack of administration of calcein or other fluorochromes before sacrifice. Indeed, withouth these markers of mineralization, it is impossible to assess dynamic histomorphometry and extent of bone formation. Also, it was not possible to adjust bone composition for tissue age as well as determined whether the observed improvement with exenatide was due to a bone matrix of better quality laid down during exenatide treatment or to amelioration of bone matrix made before exenatide administration.

Recent interest in marrow adipose tissue has revealed an unexpected role of marrow adipocyte in bone physiology. Interestingly and in contrast with previous published studies of high fat diet in the C57BL/6 congenic mouse strain (48, 49), marrow adipose volume and the number of marrow adipocytes were unchanged in HFD mice treated with saline. Others have clearly highlighted previously that mouse strain, gender and age of initiation of high fat diet likely play important role in determining the effect of HFD on bone marrow composition and skeletal phenotype (50, 51). The reason for the lack of increase in marrow adiposity in HFD + saline mice in the present study is unknown. However, it is worth noting that in humans, T2DM is not associated with an increase in marrow adipose tissue but rather marrow adipose tissue was positively correlated with HbA1c level (52). However, administration of exenatide in high fat fed animals resulted in changes in marrow adipose tissue. Marrow adipocytes and osteoblasts derived both from the same common non-hematopoietic non-endothelial CD45^−^ CD31^−^ Sca1^+^ CD24^+^ mesenchymal stem cells (53). Previous studies have highlighted the complex action of GLP-1 and its analogs on bone marrow mesenchymal stem cells with apparent higher proliferation of uncommitted progenitors resulting in higher osteoblast and marrow adipocyte numbers (54). Our results support these observations as the number of osteoblasts and marrow adipocytes were both elevated in response to exenatide. However, adipocyte-committed precursor cells are suspected to restrain osteoblast differentiation and bone formation. Adipogenic precursor cells express high level of the dipeptidyl peptidase-4 and their negative action on bone formation is alleviated by administration of a dpp-4 inhibitor (53). However, as exenatide is designed to be dpp-4 resistant, it is possible that in our study, this drug was unaffected by dpp-4 overexpression.

In conclusion, the present study provides new insight into the mode of action of exenatide on bone in diabetes. Indeed, not only did this agent ameliorated cortical bone microarchitecture but it also led to critical changes in bone composition that resulted in improvement of overall bone mechanical properties. Further studies are required to ascertain whether such effects occur in humans, but the results of this study are highly encouraging to the goal of improving bone mechanical properties in type-2 diabetes.

## Author Contributions

PF, NI, DC, and GM conceived and planned the experiments. SM and AM carried out the experiments. SM, PF, DC, NI, and GM contributed to the interpretation of the results. GM took the lead in writing the manuscript. All authors provided critical feedback and helped shape the research, analysis and manuscript.

### Conflict of Interest Statement

The authors declare that the research was conducted in the absence of any commercial or financial relationships that could be construed as a potential conflict of interest.
